# Surveillance of prognostic risk factors in patients with SCCB using artificial intelligence: a retrospective study

**DOI:** 10.1038/s41598-023-35761-w

**Published:** 2023-05-30

**Authors:** Chenghao Zhanghuang, Zhaoxia Zhang, Jinkui Wang, Zhigang Yao, Fengming Ji, Chengchuang Wu, Jing Ma, Zhen Yang, Yucheng Xie, Haoyu Tang, Bing Yan

**Affiliations:** 1 Department of Urology, Kunming Children’s Hospital (Children’s Hospital Affiliated to Kunming Medical University), Yunnan Province Clinical Research Center for Children’s Health and Disease, 288 Qianxing Road, Kunming, 650228 Yunnan People’s Republic of China; 2 Department of Urology, Chongqing Key Laboratory of Children Urogenital Development and Tissue Engineering, Chongqing Key Laboratory of Pediatrics, Ministry of Education Key Laboratory of Child Development and Disorders, National Clinical Research Center for Child Health and Disorders, China International Science and Technology Cooperation Base of Child Development and Critical Disorders, Children’s Hospital of Chongqing Medical University, Chongqing, People’s Republic of China; 3 Yunnan Key Laboratory of Children’s Major Disease Research, Yunnan Province Clinical Research Center for Children’s Health and Disease, Yunnan Clinical Medical Center for Pediatric Disease, Kunming Children’s Hospital (Children’s Hospital Affiliated to Kunming Medical University), Kunming, People’s Republic of China; 4 Department of Otolaryngology, Kunming Children’s Hospital (Children’s Hospital Affiliated to Kunming Medical University), Kunming, People’s Republic of China; 5 Department of Oncology, Yunnan Children Solid Tumor Treatment Center, Kunming Children’s Hospital (Children’s Hospital Affiliated to Kunming Medical University), Kunming, People’s Republic of China; 6 Department of Pathology, Kunming Children’s Hospital (Children’s Hospital Affiliated to Kunming Medical University), Kunming, People’s Republic of China

**Keywords:** Cancer therapy, Oncology, Bladder cancer

## Abstract

Small cell carcinoma of the bladder (SCCB) is a rare urological tumor. The prognosis of SCCB is abysmal. Therefore, this study aimed to construct nomograms that predict overall survival (OS) and cancer-specific survival (CSS) in SCCB patients. Information on patients diagnosed with SCCB during 2004–2018 was obtained from the Surveillance, Epidemiology, and End Results (SEER) database. Univariate and multivariate Cox regression models analyzed Independent risk factors affecting patients' OS and CSS. Nomograms predicting the OS and CSS were constructed based on the multivariate Cox regression model results. The calibration curve verified the accuracy and reliability of the nomograms, the concordance index (C-index), and the area under the curve (AUC). Decision curve analysis (DCA) assessed the potential clinical value. 975 patients were included in the training set (N = 687) and the validation set (N = 288). Multivariate COX regression models showed that age, marital status, AJCC stage, T stage, M stage, surgical approach, chemotherapy, tumor size, and lung metastasis were independent risk factors affecting the patients' OS. However, distant lymph node metastasis instead AJCC stage is the independent risk factor affecting the CSS in the patients. We successfully constructed nomograms that predict the OS and CSS for SCCB patients. The C index of the training set and the validation set of the OS were 0.747 (95% CI 0.725–0.769) and 0.765 (95% CI 0.736–0.794), respectively. The C index of the CSS were 0.749 (95% CI 0.710–0.773) and 0.786 (95% CI 0.755–0.817), respectively, indicating that the predictive models of the nomograms have excellent discriminative power. The calibration curve and the AUC also show good accuracy and discrimination of the nomograms. To sum up, We established nomograms to predict the OS and CSS of SCCB patients. The nomograms have undergone internal cross-validation and show good accuracy and reliability. The DCA shows that the nomograms have an excellent clinical value that can help doctors make clinical-assisted decision-making.

## Introduction

Bladder cancer (BCa) is one of the most common malignancies in the urinary system and the fifth most common globally, with about 200,000 bladder cancer deaths yearly ^1^. According to GLOBOCAN, about 573,278 new bladder cancer cases and 212,536 deaths were reported annually ^2^. According to the 2004 WHO urinary tract tumor classification criteria, bladder cancer includes urothelial (transitional cell) carcinoma, squamous cell carcinoma and adenoid-cell carcinoma, neuroendocrine tumor (such as small cell carcinoma), carcinoma of monotype, mixed carcinoma, sarcoma carcinoma, and metastatic cancer, etc. Among them, small cell carcinoma of the bladder (SCCB) is a poorly differentiated and highly aggressive neuroendocrine tumor^3^. The incidence of SCCB is extremely low, accounting for less than 1% of bladder malignancies^4^, due to the small number of cases, causing a vague understanding of the disease. The prognosis for SCCB is inferior, with a median survival of between 1 and 2 years, and untreated patients can survive for as little as 4 to 5 months^5^. Because SCCB is relatively rare, it is poorly understood, and there are still some problems in assessing its risk factors.

In recent years, the assessment of cancer risk factors has facilitated the management of cancer patients and assisted physicians in clinically assisted decision-making. Currently, the more recognized method of assessing cancer risk factors is the AJCC staging system. Still, the AJCC staging system needs more critical clinicopathological information related to patient prognosis, including treatment measures and basic patient information, so the AJCC staging system has certain limitations. The nomogram is a graphical calculation scale of the prediction model, which includes the patient's clinical characteristics and treatment methods, to maximize the prediction accuracy^6^. Nomograms of cancer have been established, including bladder cancer. Wang et al. established a nomogram that predicted the overall survival of bladder cancer patients^7^. Huang et al. developed a nomogram that can predict cancer-specific survival (CSS) in patients with SCCB, which is better than conventional AJCC staging systems^8^. However, a single institution can barely have sufficient possibilities for predictive model construction due to the low incidence of small cell bladder and the small number of cases.

The SEER database is the largest cancer database in the United States, which registers patient data from 18 cancer centers, covering more than 30% of the population, and gender, age, marital status, TNM stage, Grade grade, and patient treatment method are included in the SEER database. Therefore, many researchers collect patient information from the SEER database to establish corresponding nomograms. However, only Dong et al. reported a nomogram of SCCB in 2018^9^, and their study included 582 patients in the SEER database between 2004 and 2014, establishing the nomograms predicting CSS and OS at 1 and 3 years. However, the OS and CSS C index in this study did not exceed 0.75. The number of SCCBs has also increased over time. We wanted to re-establish the nomogram of SCCB based on the updated data and to perform multiple validations of the prediction model using the C index, calibration curve, and AUC. Therefore, this study aims to collect data on SCCB from 2004 to 2018 in the SEER database, develop nomograms that can predict patient OS and CSS, and evaluate the potential clinical value of the prediction model by time-based DCA curves. In addition, we wanted to perform a KM curve analysis of independent risk factors separately based on data from COX multivariate regression models to figure out the effect of each independent risk factor on OS and CSS in patients with SCCB.

## Materials and methods

### Data source and extraction

Patient information was obtained from the SEER database, and patients diagnosed with SCCB between 2008 and 104 were included in this study. This study counted the number of bladder cancer and the proportion of SCCB between 2004 and 2018 (Fig. S1). The SEER database is a national cancer database registering data from multiple cancer centers, and this study contains a total of 18 cancer centers, covering about 30% of the population. Data in the SEER database are publicly available, and patient information is anonymized. This study followed the guidelines published by the SEER database and did not require ethical approval and patient-informed consent.

**Figure 1:**
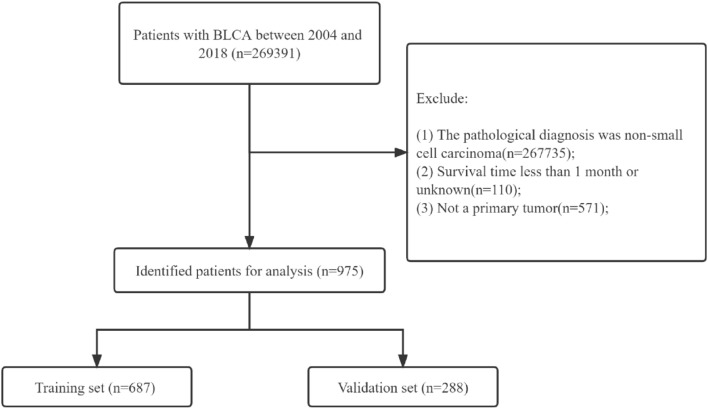
Flowchart for inclusion and exclusion of patients with SCCB.

This study collected all clinicopathological information, including age, marital status, tumor size, grade, TNM stage, sex, and ethnicity. Furthermore, treatment modalities, including surgery, radiotherapy, and chemotherapy, are also included. In addition, we had follow-up outcomes, including survival status, survival time, and cause of death. The inclusion criteria for this study: Patients with a pathologic diagnosis of SCCB.The exclusion criteria for this study included: (1) The pathological diagnosis of non-small-cell bladder cancer. (2) The survival time is less than 1 month, or the survival time is unknown. (3) It is not the primary tumor. The flowchart of the data screening in this study is shown in Fig. 1.

### Development and validation

All patients were randomized into training and validation sets in a 7:3 ratio for nomogram development and internal validation. Univariate and multivariate COX regression models were used to analyze the training set's independent risk factors affecting patient OS and CSS. Then, nomograms were constructed using independent risk factors based on a multivariate COX regression model and were used to predict the OS and CSS of SCCB at 1-, 3-, and 5 years, respectively. 1000 bootstrap samples were used to detect the model accuracy. The consistency index (C-index) and area under the receiver operating characteristic curve (AUC) are used to verify the identification ability of the models.

### Clinical application

The decision analysis curve (DCA) is used to test the potential clinical value of the predictive models. Furthermore, we also calculated the risk values for each patient. The cutoff value of the risk value was also determined using the receiver operating characteristic curve (ROC). All patients using cutoffs were divided into high-risk and low-risk groups. The Kaplan–Meier (K–M) curves and the log-rank test compared the OS and CSS differences between the high and low-risk groups. In addition, we also analyzed the survival differences between the different tumor sizes, T stages, surgical procedures, chemotherapy approaches, and the risks of metastasis in all patients.

### Statistical analysis

Continuous variables like age were described using the mean and the standard deviation. Group comparisons were made using the chi-square test or non-parametric U test. Categorical variables such as tumor size, stage, and surgical method were described by frequency and compared using the chi-square test. A Cox regression model analyzed prognostic influencing factors of the patients, and the log-rank test tested the patient survival differences. Statistical analysis was performed using the R software version 4.1.0 and SPSS 26.0. The R packages used included "DynNom", "RMS", "Survival", and "ggDCA". p values less than 0.05 were considered statistically significant.

### Informed consent

This study is accordance with relevant guidelines and regulations. All the data in our study were obtained from the SEER database. This is a publicly open database and does not require informed consent from the subjects and/or their legal guardians.

## Results

### Clinical features

In total, 975 patients were enrolled in this study according to the inclusion–exclusion criteria. The training and validation sets were 687 and 288 cases, respectively. The mean age of the patients was 71.2 ± 11.4 years old, and the patients were predominantly male (75.4%). The patients' race was mostly white, 89.6%, 0.57% married and 43% unmarried. No significant difference in the primary site of the tumor. The Grade grades I–III were 22.5%, grade IV 36%, and grades unknown 41.5%. AJCC stage I was 6.77%, II at 23.9%, III at 8.21%, IV at 25.7%, and AJCC stage was unknown at 35.4%. The T stage was mainly observed in T2, accounting for 51%. The M stage was mainly M0, accounting for 75.7%. N staging is dominated by N0, accounting for 71.6%. The procedure included local tumor excision (63.5%), radical cystectomy (27%), no surgery (9.03%), and unknown (0.51%). The total number of patients receiving chemotherapy was 67.3%. Patients receiving radiotherapy accounted for 28.4%. The tumor size was mainly ≤ 5 cm, accounting for 41.4%. Patients with bone metastases were 7.18%, 9.44% with liver metastases, 4.41% with lung metastases, and 16.9% with distant lymph node metastases. There were no significant differences between the training and validation sets; details are shown in Table 1.

**Table 1:** Clinicopathological characteristics of patients with SCCB.

	All	Training cohort	Validation cohort	p
	N = 975	N = 687	N = 288	
Age	71.2 (11.4)	71.1 (11.2)	71.2 (11.8)	0.888
Sex				0.580
Male	735 (75.4%)	514 (74.8%)	221 (76.7%)	
Female	240 (24.6%)	173 (25.2%)	67 (23.3%)	
Race				0.532
White	874 (89.6%)	612 (89.1%)	262 (91.0%)	
Black	56 (5.74%)	40 (5.82%)	16 (5.56%)	
Other	45 (4.62%)	35 (5.09%)	10 (3.47%)	
Marital				0.858
No/unknown	419 (43.0%)	297 (43.2%)	122 (42.4%)	
Married	556 (57.0%)	390 (56.8%)	166 (57.6%)	
Primary site				0.469
Trigone of bladder	50 (5.13%)	41 (5.97%)	9 (3.12%)	
Dome of bladder	71 (7.28%)	49 (7.13%)	22 (7.64%)	
Lateral wall of bladder	202 (20.7%)	155 (22.6%)	47 (16.3%)	
Anterior wall of bladder	54 (5.54%)	40 (5.82%)	14 (4.86%)	
Posterior wall of bladder	96 (9.85%)	51 (7.42%)	45 (15.6%)	
Bladder neck	16 (1.64%)	12 (1.75%)	4 (1.39%)	
Overlapping lesion of bladder	151 (15.5%)	101 (14.7%)	50 (17.4%)	
Bladder, NOS	335 (34.4%)	238 (34.6%)	97 (33.7%)	
Grade				0.061
I–III	219 (22.5%)	159 (23.1%)	60 (20.8%)	
IV	351 (36.0%)	254 (37.0%)	97 (33.7%)	
Unknown	405 (41.5%)	274 (39.9%)	131 (45.5%)	
AJCC stage				0.566
I	66 (6.77%)	46 (6.70%)	20 (6.94%)	
II	233 (23.9%)	163 (23.7%)	70 (24.3%)	
III	80 (8.21%)	51 (7.42%)	29 (10.1%)	
IV	251 (25.7%)	175 (25.5%)	76 (26.4%)	
Unknown	345 (35.4%)	252 (36.7%)	93 (32.3%)	
T				0.662
≤ T1	143 (14.7%)	101 (14.7%)	42 (14.6%)	
T2	497 (51.0%)	352 (51.2%)	145 (50.3%)	
T3	145 (14.9%)	95 (13.8%)	50 (17.4%)	
T4	115 (11.8%)	84 (12.2%)	31 (10.8%)	
Tx	75 (7.69%)	55 (8.01%)	20 (6.94%)	
M				0.500
M0	738 (75.7%)	525 (76.4%)	213 (74.0%)	
M1	230 (23.6%)	158 (23.0%)	72 (25.0%)	
Mx	7 (0.72%)	4 (0.58%)	3 (1.04%)	
N				0.418
N0	698 (71.6%)	490 (71.3%)	208 (72.2%)	
N1	83 (8.51%)	55 (8.01%)	28 (9.72%)	
N2	97 (9.95%)	66 (9.61%)	31 (10.8%)	
N3	24 (2.46%)	19 (2.77%)	5 (1.74%)	
Nx	73 (7.49%)	57 (8.30%)	16 (5.56%)	
Surgery				0.583
No	88 (9.03%)	64 (9.32%)	24 (8.33%)	
Local tumor excision	619 (63.5%)	436 (63.5%)	183 (63.5%)	
Radical cystectomy	263 (27.0%)	182 (26.5%)	81 (28.1%)	
Unknown	5 (0.51%)	5 (0.73%)	0 (0.00%)	
Chemotherapy				0.192
No/unknown	319 (32.7%)	234 (34.1%)	85 (29.5%)	
Yes	656 (67.3%)	453 (65.9%)	203 (70.5%)	
Radiation				0.232
No/unknown	698 (71.6%)	500 (72.8%)	198 (68.8%)	
Yes	277 (28.4%)	187 (27.2%)	90 (31.2%)	
Tumor size (cm)				0.286
≤ 5	404 (41.4%)	291 (42.4%)	113 (39.2%)	
> 5	235 (24.1%)	156 (22.7%)	79 (27.4%)	
Unknown	336 (34.5%)	240 (34.9%)	96 (33.3%)	
Mets at DX bone				0.554
Yes	70 (7.18%)	52 (7.57%)	18 (6.25%)	
No/unknown	905 (92.8%)	635 (92.4%)	270 (93.8%)	
Mets at DX liver				0.750
Yes	92 (9.44%)	63 (9.17%)	29 (10.1%)	
No/unknown	883 (90.6%)	624 (90.8%)	259 (89.9%)	
Mets at DX lung				0.785
Yes	43 (4.41%)	29 (4.22%)	14 (4.86%)	
No/unknown	932 (95.6%)	658 (95.8%)	274 (95.1%)	
Mets at distant LN				0.146
Yes	165 (16.9%)	108 (15.7%)	57 (19.8%)	
No/unknown	810 (83.1%)	579 (84.3%)	231 (80.2%)	
CSS				0.762
Dead	581 (59.6%)	412 (60.0%)	169 (58.7%)	
Alive	394 (40.4%)	275 (40.0%)	119 (41.3%)	
Survival months	25.4 (34.5)	25.4 (35.0)	25.4 (33.1)	1.000
OS				0.921
Dead	708 (72.6%)	500 (72.8%)	208 (72.2%)	
Alive	267 (27.4%)	187 (27.2%)	80 (27.8%)	

### Univariate and multivariate COX regression analysis

This study used a univariate COX regression model to screen out the relevant factors affecting both OS and CSS in the training set. The results showed that the factors affecting the OS in the patients in the training set included age, marital status, the primary part of the tumor, AJCC stage, TNM stage, surgery, chemoradiotherapy, tumor size, and bone liver lung metastasis and distant lymph node metastasis. Age, marital status, AJCC stage, T stage, M stage, surgical mode, chemotherapy, tumor size, and lung metastasis were the independent risk factors for the patient's OS. Factors influencing CSS in patients in the training set included age, marital status, primary tumor site, AJCC stage, TNM stage, surgery, chemotherapy, tumor size, bone, liver, and lung metastasis, and distant lymph node metastasis. Among them, the primary tumor site, AJCC stage, N stage, bone metastasis, and liver metastasis were not independent risk factors affecting CSS. Detailed results are presented in Tables 2 and 3.

**Table 2:** Univariate and multivariate analyses of 0S in training cohort.

	Univariate			Multivariate		
	HR	95%CI	p	HR	95%CI	p
Age	1.04	1.03–1.05	< 0.001	1.03	1.021–1.039	< 0.001
Sex						
Male						
Female	0.98	0.80–1.20	0.863			
Race						
White						
Black	1.35	0.95–1.93	0.095			
Other	0.81	0.53–1.22	0.312			
Marital						
No/unknown						
Married	0.65	0.54–0.77	< 0.001	0.653	0.545–0.783	< 0.001
Primary site						
Trigone of bladder						
Dome of bladder	0.93	0.57–1.52	0.763			
Lateral wall of bladder	0.83	0.55–1.26	0.39			
Anterior wall of bladder	0.59	0.34–1.02	0.061			
Posterior wall of bladder	0.93	0.57–1.51	0.771			
Bladder neck	2.47	1.25–4.89	0.009			
Overlapping lesion of bladder	0.96	0.62–1.47	0.835			
Bladder, NOS	1.01	0.68–1.5	0.965			
Grade						
I–III						
IV	1.02	0.82–1.28	0.844			
Unknown	1.13	0.9–1.43	0.278			
AJCC stage						
I						
II	1.09	0.69–1.73	0.707	1.167	0.66–2.063	0.595
III	1.3	0.75–2.26	0.35	1.444	0.743–2.803	0.278
IV	2.57	1.65–3.99	< 0.001	1.873	1.065–3.293	0.029
Unknown	1.62	1.05–2.49	0.029	1.282	0.767–2.143	0.344
T						
≤ T1						
T2	0.94	0.72–1.22	0.642	1.019	0.729–1.424	0.911
T3	1.25	0.9–1.73	0.177	1.51	0.992–2.298	0.055
T4	1.51	1.07–2.12	0.019	1.671	1.108–2.519	0.531
Tx	2.78	1.92–4	< 0.001	1.135	0.764–1.685	0.014
M						
M0						
M1	2.5	2.04–3.05	< 0.001	1.703	1.281–2.264	< 0.001
Mx	2.34	0.87–6.27	0.092	1.337	0.484–3.692	0.575
N						
N0						
N1	1.42	1.03–1.96	0.032			
N2	1.51	1.13–2.01	0.005			
N3	1.43	0.82–2.5	0.204			
Nx	1.99	1.47–2.68	< 0.001			
Surgery						
No						
Local tumor excision	0.54	0.41–0.71	< 0.001	1.083	0.788–1.489	0.621
Radical cystectomy	0.31	0.23–0.43	< 0.001	0.756	0.512–1.115	0.159
Unknown	1	0.4–2.49	0.998	2.322	0.9–5.995	0.012
Chemotherapy						
No/unknown						
Yes	0.48	0.4–0.57	< 0.001	0.529	0.429–0.651	< 0.001
Radiation						
No/unknown						
Yes	0.81	0.67–0.99	0.044			
Tumor size (cm)						
≤ 5						
> 5	1.74	1.39–2.19	< 0.001	1.496	1.18–1.897	0.001
Unknown	1.66	1.35–2.03	< 0.001	1.33	1.077–1.643	0.008
Mets at DX bone						
Yes						
No/unknown	0.51	0.37–0.7	< 0.001			
Mets at DX liver						
Yes						
No/unknown	0.39	0.3–0.52	< 0.001			
Mets at DX lung						
Yes						
No/unknown	0.31	0.21–0.45	< 0.001	0.639	0.42–0.973	0.037
Mets at Distant LN						
Yes						
No/unknown	0.67	0.54–0.84	0.001

**Table 3:** Univariate and multivariate analyses of CSS in training cohort.

	Univariate			Multivariate		
	HR	95%CI	p	HR	95%CI	p
Age	1.03	1.02–1.04	< 0.001	1.022	1.012–1.031	< 0.001
Sex						
Male						
Female	1.08	0.87–1.35	0.468			
Race						
White						
Black	1.35	0.92–1.99	0.123			
Other	0.78	0.49–1.25	0.303			
Marital						
No/unknown						
Married	0.66	0.55–0.81	< 0.001	0.688	0.563–0.841	< 0.001
Primary site						
Trigone of bladder						
Dome of bladder	0.81	0.47–1.4	0.457			
Lateral wall of bladder	0.81	0.52–1.27	0.358			
Anterior wall of bladder	0.65	0.37–1.15	0.14			
Posterior wall of bladder	0.88	0.52–1.49	0.63			
Bladder neck	2.23	1.06–4.68	0.034			
Overlapping lesion of bladder	0.85	0.53–1.37	0.511			
Bladder, NOS	0.93	0.6–1.43	0.741			
Grade						
I–III						
IV	1.02	0.8–1.31	0.848			
Unknown	1.13	0.88–1.45	0.339			
AJCC stage						
I						
II	1.09	0.65–1.83	0.748			
III	1.41	0.77–2.6	0.27			
IV	2.92	1.78–4.78	< 0.001			
Unknown	1.7	1.05–2.77	0.032			
T						
≤ T1						
T2	0.99	0.73–1.34	0.954	1.12	0.821–1.529	0.474
T3	1.32	0.92–1.89	0.13	1.739	1.158–2.611	0.008
T4	1.67	1.14–2.43	0.008	1.637	1.066–2.513	0.398
Tx	2.86	1.9–4.28	< 0.001	1.193	0.793–1.795	0.024
M						
M0						
M1	2.84	2.29–3.51	< 0.001	2.187	1.674–2.858	< 0.001
Mx	2.82	1.05–7.57	0.04	1.863	0.672–5.166	0.232
N						
N0						
N1	1.67	1.2–2.34	0.002			
N2	1.68	1.24–2.28	0.001			
N3	1.59	0.89–2.85	0.116			
Nx	2.16	1.56–3	< 0.001			
Surgery						
No						
Local tumor excision	0.51	0.38–0.68	< 0.001	0.969	0.689–1.363	0.857
Radical cystectomy	0.28	0.2–0.4	< 0.001	0.675	0.437–1.042	0.076
Unknown	1.1	0.44–2.75	0.842	2.85	1.085–7.484	0.033
Chemotherapy						
No/unknown						
Yes	0.49	0.4–0.6	< 0.001	0.516	0.414–0.643	< 0.001
Radiation						
No/unknown						
Yes	0.8	0.65–1	0.054			
Tumor size (cm)						
≤ 5						
> 5	1.87	1.46–2.4	< 0.001	1.503	1.157–1.952	0.002
Unknown	1.73	1.38–2.17	< 0.001	1.318	1.042–1.668	0.021
Mets at DX bone						
Yes						
No/unknown	0.46	0.33–0.63	< 0.001			
Mets at DX liver						
Yes						
No/unknown	0.36	0.27–0.48	< 0.001			
Mets at DX lung						
Yes						
No/unknown	0.31	0.2–0.46	< 0.001	0.627	0.402–0.977	0.039
Mets at distant LN						
Yes						
No/unknown	0.6	0.47–0.76	< 0.001	0.669	0.515–0.871	0.003

### Development and validation of the nomograms

**Figure 2:**
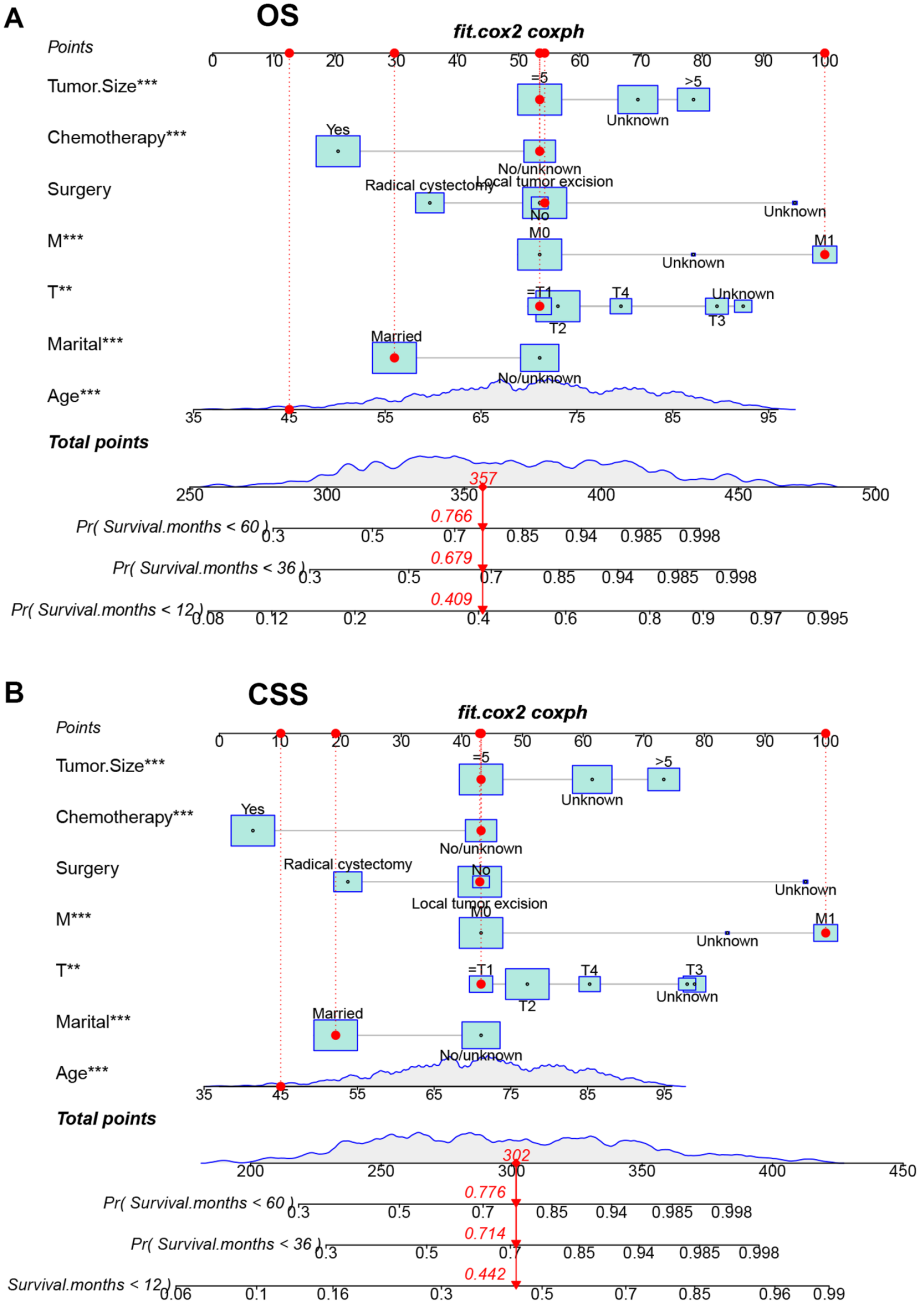
The nomograms for predicting 1-, 3-, 5-year OS and CSS in SCCB patients. (**A**) The nomogram for predicting OS. (**B**) The nomogram for predicting CSS.

We developed two new nomograms based on the results of the multivariate COX regression models used to predict patients ' years of OS and CSS (Fig. 2). The nomogram of the OS showed that the tumor size, surgical approach, chemotherapy, distant metastasis, and T stage were the most significant influencing factors for the OS. In addition, marital status and age can also affect the OS. The nomogram of the CSS showed that the tumor size, surgery, M stage, and T stage were the most significant influencing factors for the CSS. In addition, chemotherapy, marital status, and age can affect CSS.

**Figure 3:**
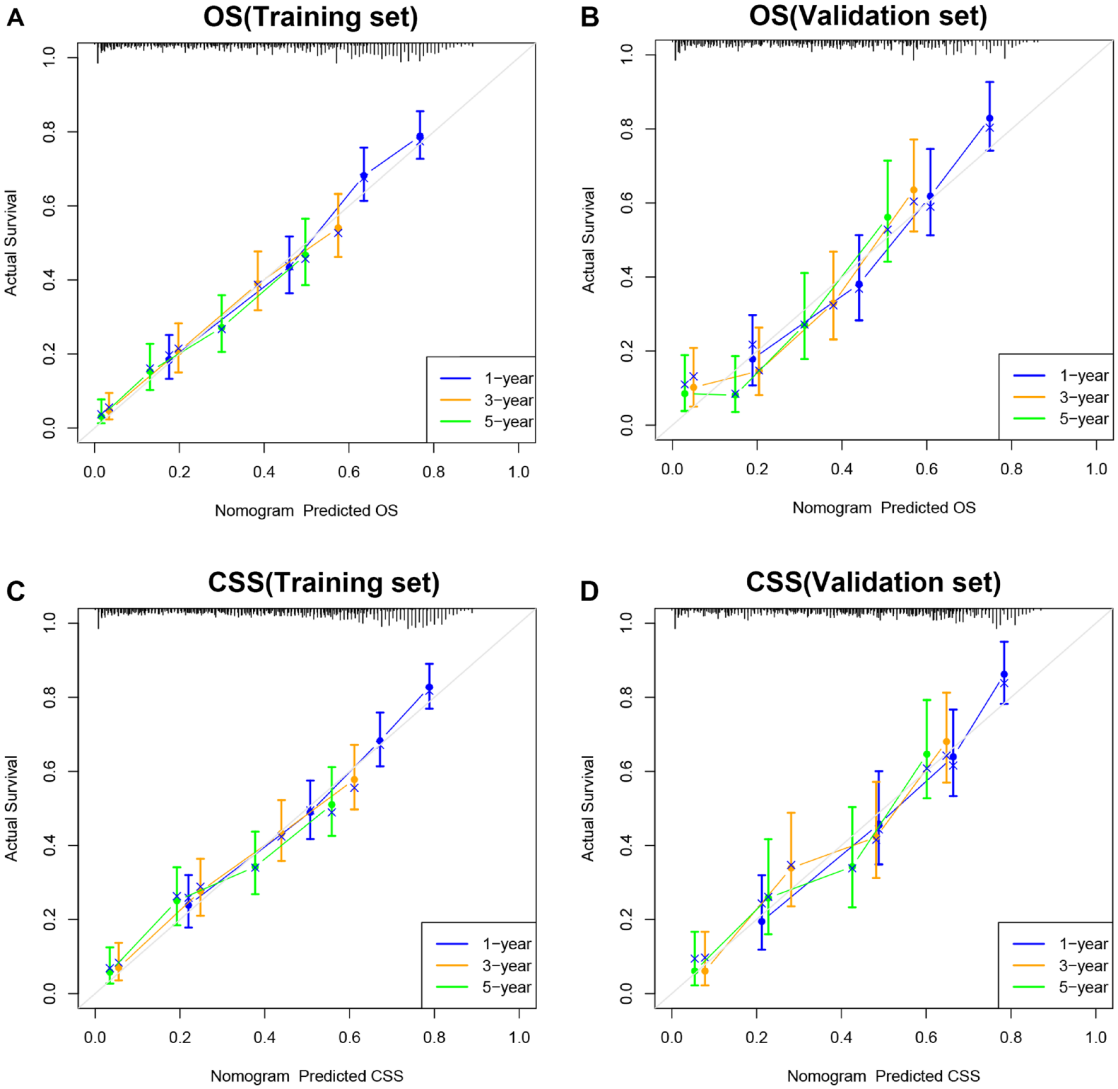
Calibration curve of the nomograms for predicting 1-, 3-, 5-year OS and CSS in SCCB patients. (**A**) Calibration curve of the nomograms for predicting OS in the training set. (**B**) Calibration curve of the nomograms for predicting OS in the validation set. (**C**) Calibration curve of the nomograms for predicting CSS in the training set. (**D**) Calibration curve of the nomograms for predicting CSS in the validation set. The horizontal axis is the predicted value in the nomogram, and the vertical axis is the observed value.

**Figure 4:**
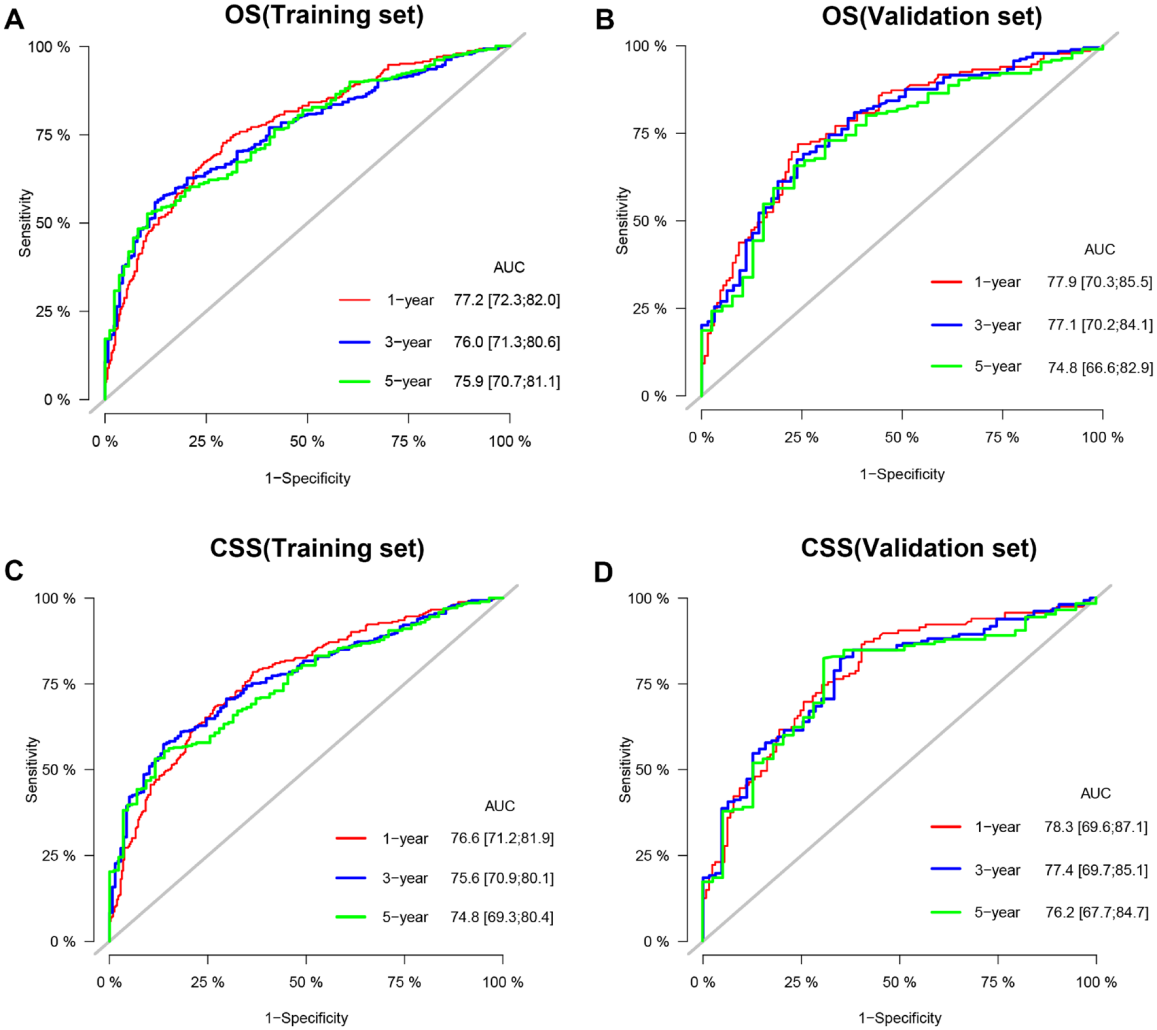
AUC for predicting 1-, 3-, and 5-year OS and CSS in SCCB patients. (**A**) The AUC for OS in the training set. (**B**) The AUC for OS in the validation set. (**C**) The AUC for CSS in the training set. (**D**) The AUC for CSS in the validation set.

Furthermore, we used internal validation to validate the model's accuracy and discriminability. The C index of the OS in the training set and the validation set were 0.747 (95% CI 0.725–0.769) and 0.765 (95% CI 0.736–0.794, respectively). The C index of the CSS in the training set and the validation set were 0.749 (95% CI 0.710–0.773) and 0.786 (95% CI 0.755–0.817), respectively, which showed that the nomograms of both the OS and CSS have good recognition ability. In addition, the calibration curve shows that the predicted values of the OS and CSS nomogram prediction models are highly consistent with the actual observed values (Fig. 3). The AUC results showed good discriminability of the nomograms, the OS in training set with an AUC of 77.2, 76.0, 75.9 at 1-,3-, and 5 years, respectively. The AUC for OS in the validation was 77.9, 77.1, 74.8, respectively. AUC for CSS in the training set was 76.6, 75.6, 74.8, respectively, and AUC for CSS was 78.3, 77.4, 76.2 in the validation set (Fig. 4).

### Clinical application of the nomograms

**Figure 5:**
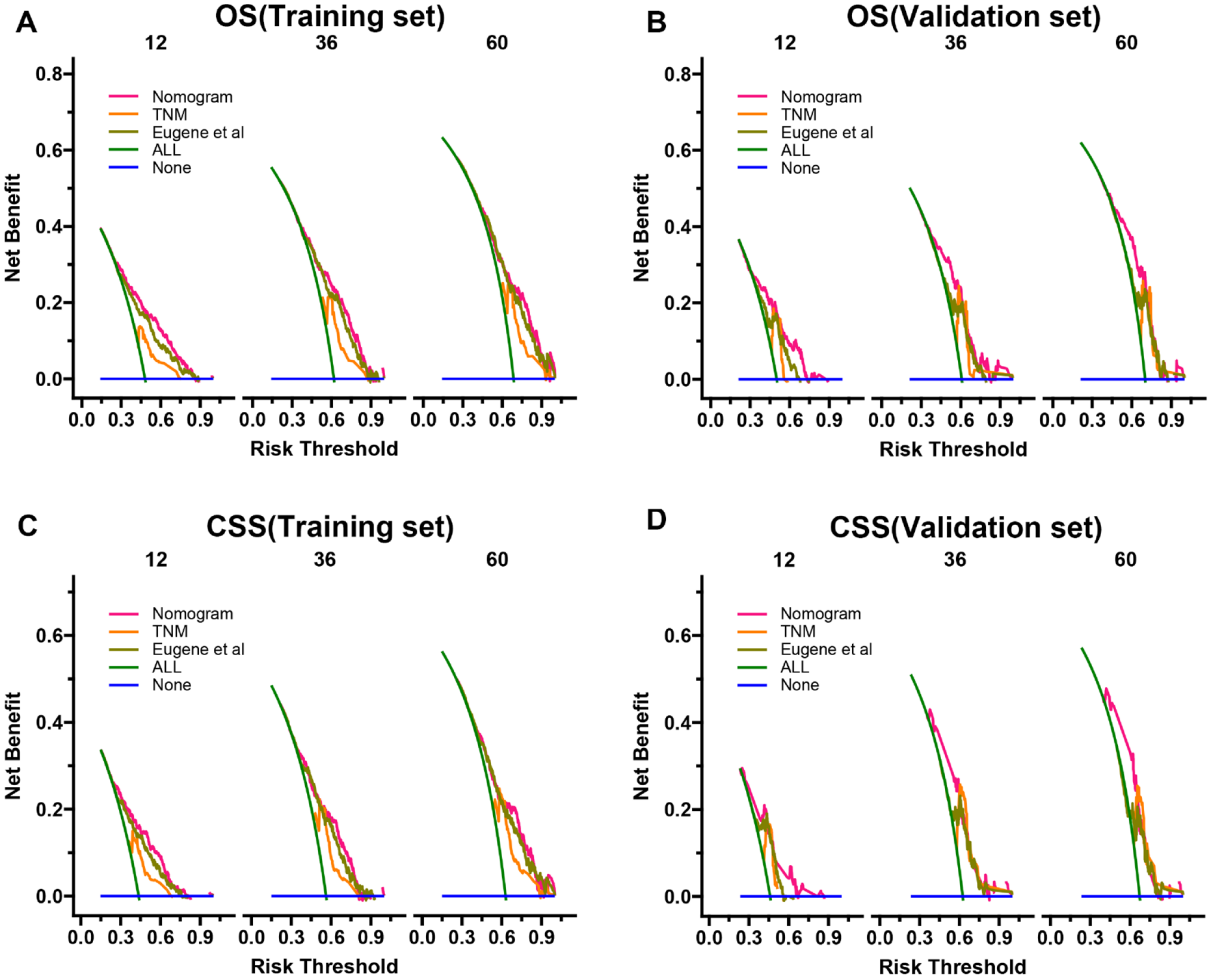
DCA of the nomograms for predicting OS and CSS. (**A**) The nomogram for OS in the training set. (**B**) The nomogram for OS in the validation set. (**C**) The nomogram for CSS in the training and training set. (**D**) The nomogram for CSS in the training and validation set.

**Figure 6:**
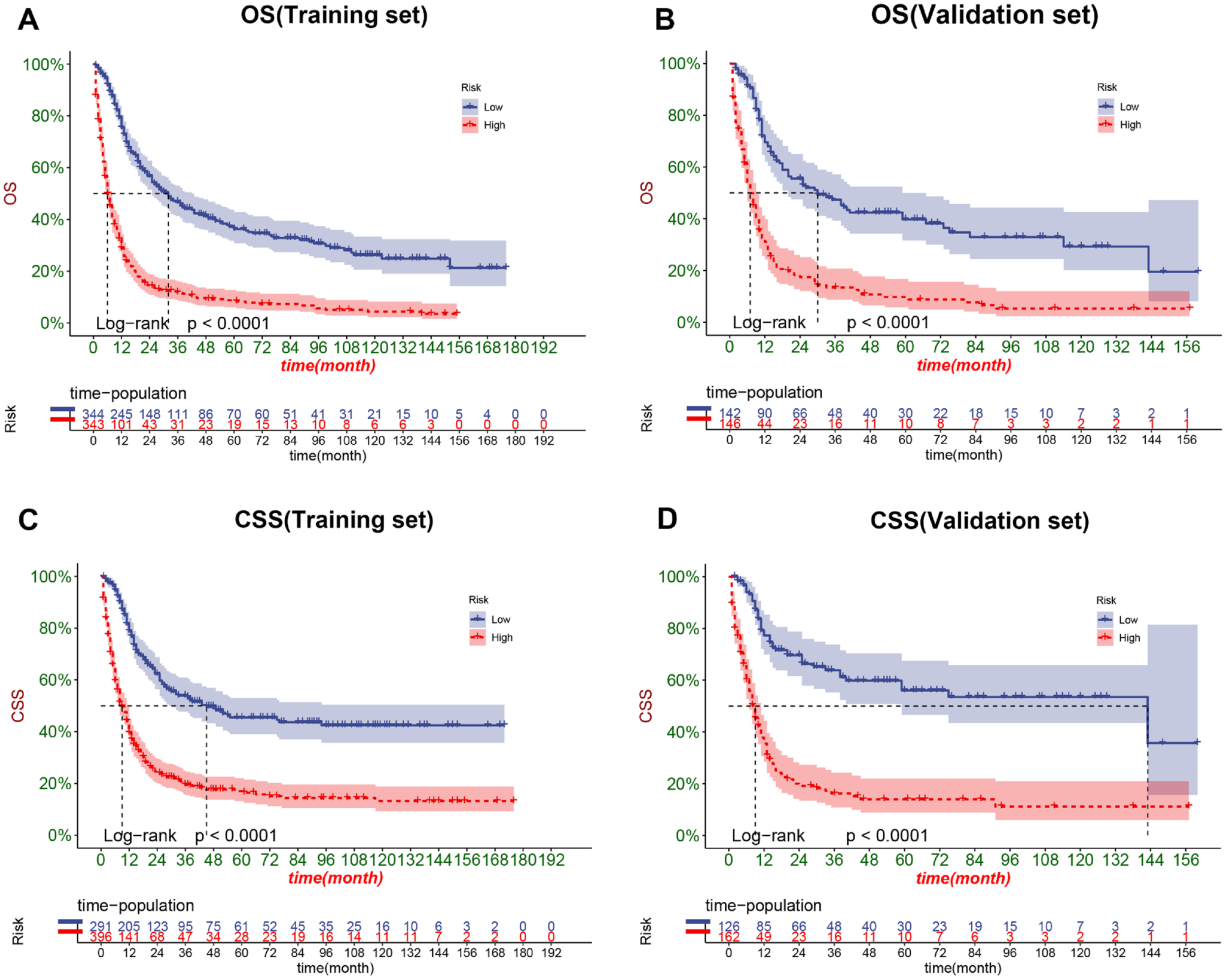
Kaplan–Meier curves of patients in the low-risk and high-risk groups.

**Figure 7:**
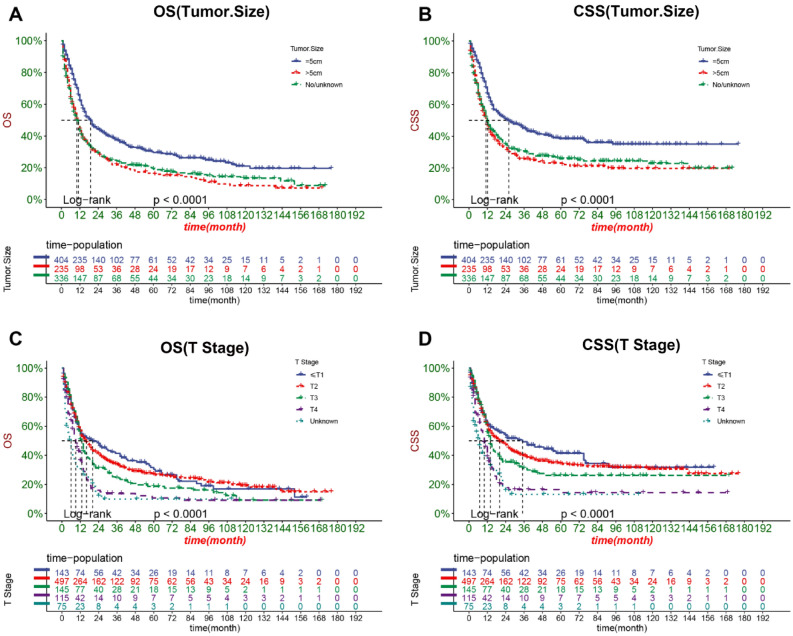
Kaplan–Meier curves of patients with different tumor size and T stage. (**A**) The OS rate of patients with different tumor size. (**B**) The CSS rate of patients with different tumor size. (**C**) The OS rate of patients with different T stage. (**D**) The CSS rate of patients with different T stage.

**Figure 8:**
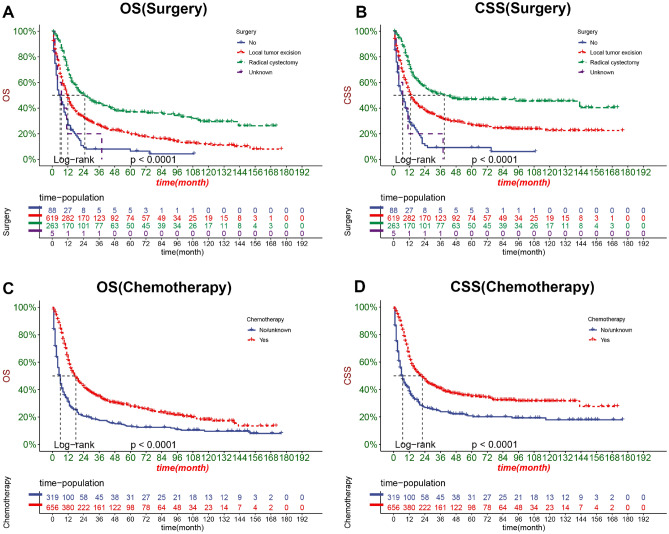
Kaplan–Meier curves of patients with surgery and chemotherapy. (**A**) The OS rate of patients with different surgery. (**B**) The CSS rate of patients with different surgery. (**C**) The OS rate of patients with or without chemotherapy. (**D**) The CSS rate of patients with or without chemotherapy.

**Figure 9:**
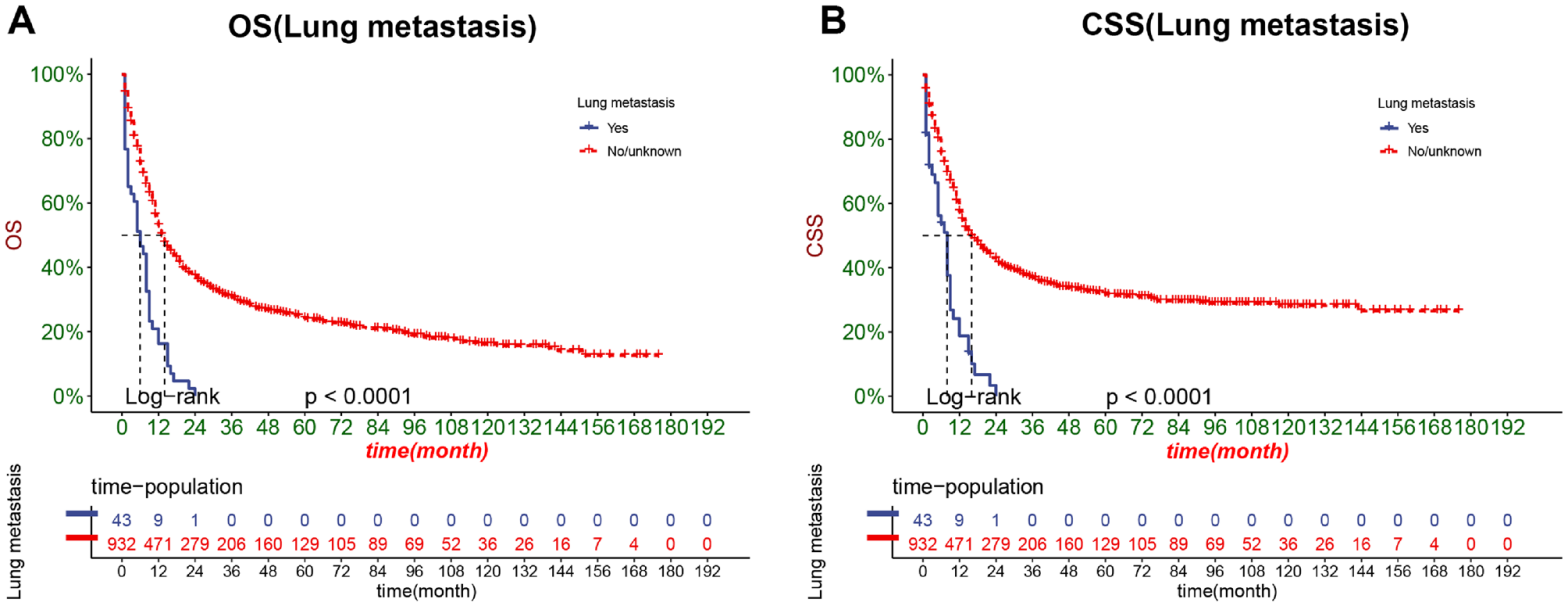
Kaplan–Meier curves of patients with lung metastases, different AJCC stage and distant lymph node metastasis. (**A**) The OS rate of patients with lung metastases. (**B**) The CSS rate of patients lung metastases.

The DCA results showed that the nomograms of both OS and CSS had good clinical value (Fig. 5). And our established OS and CSS nomograms are better than the traditional TNM staging system and the prediction model for SCCB found by Eugene et al. In addition, patients were divided into high-risk groups (total score ≥ 151.6564) and low-risk groups (total score < 151.6564) for CSS, high-risk groups (total score ≥ 151.16284) and low-risk groups(Total score < 151.16284) for OS based on the cutoff value calculated from the ROC curve. The K–M curve showed that the OS and CSS of the high-risk group were significantly lower than those of the low-risk group in both the training and validation sets (Fig. 6). The 1-, 3-, and 5-year survival rates for the high-risk group for OS were 29.45%, 12.19%, and 9.03%, respectively, and the 1-, 3-, and 5-year survival rates for the low-risk group for OS were 73.7%, 47.0%, and 37.4%, respectively. The 1-, 3-, and 5-year survival rates for the high-risk group for CSS were 38.4%, 18.5%, and 16.1%, respectively, and the 1-, 3-, and 5-year survival rates for the low-risk group for CSS were 78.3%, 57.0%, and 48.9%, respectively.In addition, the KM curve also showed that patients with tumor size ≤ 5 cm had the highest OS and CSS rates, and secondly, the lower the T stage, the higher the OS and CSS rates of the patients (Fig. 7). Patients who underwent radical cystectomy had the highest OS and CSS, followed by patients with local tumor excision, and those who received chemotherapy also had higher OS and CSS (Fig. 8). The KM curve also showed that the OS and CSS rates of patients with lung metastases were lower than those without lung metastases, and the survival time of patients with lung metastases did not exceed 24 months; the OS of patients was closely related to the AJCC stage and the lower the AJCC stage was, patients had higher OS; finally, patients with distant lymph node metastases had lower CSS (Fig. 9).

## Discussion

Primary SCCB is an extremely rare histological subtype of bladder cancer with extremely low incidence and poor prognosis. Data shows that the median survival time of SCCB patients is 12.7 months^10^. We counted the number of bladder cancer cases and the proportion of SCCB in 2004–2018 from the SEER database. We found that although the incidence of SCCB was still very low, its incidence also increased yearly. Therefore, evaluating the factors affecting the prognosis of patients with SCCB is essential. The nomogram is a tool to assess patients' survival, allowing for the cumulative effects of all prognostic factors to predict the survival probability at 1-,3-, and 5 years^6^. This study used the data from the SEER database to establish two nomograms predicting both OS and CSS in patients with primary SCCB. Our predictive model showed that age, marital status, AJCC stage, T stage, M stage, surgical mode, chemotherapy, tumor size, and lung metastasis were independent risk factors affecting the patients' OS. However, age, marital status, T stage, M stage, surgery, chemotherapy, tumor size, lung metastasis, and distant lymph node metastasis were the independent risk factors affecting the CSS in the patients.

Age is associated with the incidence and prognosis of many cancers, and studies have shown that age is an independent risk factor for cancers such as non-small cell lung cancer and breast cancer^11,12^. Previous studies showed that about 90% of bladder cancer patients are over 55 years old, and the average age at diagnosis is 73 years old^13^. The study by Feng et al. showed that younger bladder cancer patients had higher postoperative OS and CSS than older patients^14^. Several studies have reported that older bladder cancer patients have a higher mortality rate than younger patients^15^. Our results showed that the mean onset of SCCB is around 71 years old, similar to the mean age of bladder carcinoma reported by Eugene and Dong et al.^9,16^. Our prediction model showed that age was an independent risk factor for both OS and CSS in patients, with older patients having lower OS and CSS, which is consistent with the prediction model results of Dong et al.

Marital status is considered a prognostic factor in many cancers, and married status is a prognostic protective factor in most cancer patients^17^. Tao et al. found that marital status was an independent prognostic factor for OS in patients with distant metastasis of bladder cancer^18^. Sammon et al. demonstrated that marital status was a risk factor for reduced survival after radical cystectomy and that unmarried conditions impaired the prognosis of bladder cancer patients^19^. Marriage is considered a protective factor for bladder cancer, given that marriage brings patients more financial support and psychological comfort. However, the study by Dong et al. also confirmed that marital status was an independent risk factor for OS but not for CSS in patients with SCCB. Our nomogram shows that being married is a protective factor for OS and CSS in patients with SCCB, considering that the reason may be the increased number of cases.

Previous studies have shown that tumor size is a risk factor for poor prognosis in bladder cancer patients^20^. The study by Lee et al. confirmed that larger tumors in bladder cancer were significantly associated with a shorter time of recurrence^21^. The survey by Karl H Tully et al. confirmed that the smaller the tumor size of non-muscle-invasive bladder cancer, the higher the progression-free survival and CSS in bladder cancer patients^22^. The nomogram conducted by Tian et al. found that tumor size was an independent risk factor for lymph node metastasis in bladder cancer^23^. The nomogram established by Zhan showed that the tumor size was associated with the CSS prognosis in lymph node-positive bladder cancer patients^24^. Our nomogram and KM curves showed that the smaller the tumor volume of SCCB, the better the patient prognosis, consistent with Dong et al.^9^.

Currently, the treatment of bladder cancer is mainly surgery, radiotherapy, and chemotherapy. However, because the SCCB is very rare, no unified standard scheme exists for its treatment. The study by Carlo Cattrini et al. found that the surgical treatment of SCCB patients had better survival^25^. David Pasquier et al. showed that radical cystectomy does not affect DFS or OS in patients with non-metastatic SCCB and that conservative treatment is appropriate in this setting^26^. However, our nomogram showed a better prognosis for SCCB patients with radical resection, considering that the reason may be that most of the SCCBs are limited to the pelvic cavity so that curative resection can achieve a better therapeutic effect. The KM curve also showed that patients who underwent radical resection had higher OS and CSS. Considering that SCCB is mostly primary cancer, better therapeutic results can be achieved by radical resection. In addition, chemotherapy as an adjuvant therapy remains the primary treatment modality for SCCB patients^27^, and Mackey et al. reported that chemotherapy could significantly improve the prognosis of SCCB patients^28^. The study by Chau et al. found poor overall outcomes for patients with SCCB but improved survival for those who were able to receive chemotherapy^29^. Our KM curve also showed that the chemotherapy patients had a higher OS and CSS, consistent with previous studies.

Taking the traditional AJCC staging system as the standard, the AJCC staging system is based on three elements: the T stage reflects the depth of infiltration, the N stage reflects the lymph node state, and the M stage reflects the metastatic state. The nomogram of Yang et al. showed that the lower the T stage, the better the prognosis of bladder cancer patients after radical resection^30^. The N stage represents lymph node metastasis but is confined to the pelvic cavity.However, our study showed that the N stage was not an independent risk factor for OS and CSS in SCCB patients. However, the results of Eugene et al. in 2011 showed that the N stage is an independent risk factor related to the prognosis of SCCB patients^16^. Considering the highly high malignancy of SCCB, the lymph node metastasis confined to the pelvic cavity may not significantly impact its prognosis, and the result may change due to the sample size update.The critical factor affecting the prognosis of bladder cancer patients may be distant metastasis, and the nomogram of Yang et al. confirmed that distant metastasis is an important factor affecting the OS and CSS in bladder cancer patients^31^. Our nomogram also showed that SCCB patients developing distant metastases had worse OS and CSS. At the same time, distant metastases include distant lymph node metastasis and organ metastases. This study separately analyzed bone metastasis, liver metastases, lung metastasis and distant lymph node metastasis.Univariate COX regression analysis showed that both bone metastasis, liver metastasis, lung metastasis and distant lymph node metastasis were essential factors for patient OS and CSS, while the multivariate COX regression model showed that lung metastasis was a common independent risk factor for OS and CSS in SCCB patients, while distant lymph node metastasis was an independent risk factor for CSS in SCCB patients. The KM curve also showed that the patients with lung metastases had a lower OS and CSS and that most patients with lung metastases survived for no more than 24 months. At the same time, patients with SCCB with distant lymph node metastases had a lower CSS. However, the results may be biased due to the small number of organ metastasis cases.

Although the excellent accuracy of the nomogram based on the SEER database was confirmed by internal validation, crucial clinical information related to prognoses, such as smoking and BMI, still needs to be improved, thus some limitations in this study. Secondly, the studies based on the SEER database were retrospective, and selection bias cannot be avoided. Further validation through prospective studies may be required. However, many essential variables were included, so our results were not significantly biased. Moreover, there are very few prediction models for SCCB at present. This study further expanded the sample size based on the prediction model of Dong et al.^9^. Meanwhile, the C index obtained in this study is higher, showing that the accuracy of the prediction model established after expanding the sample size is increased. Meanwhile, we conducted a separate KM analysis of all the related independent risk factors, which made the prediction model more convincing. In addition, our DCA analysis included the traditional TNM stage and the prediction model of Eugene et al.^16^. The results showed the highest clinical application value of our nomogram.

## Conclusions

This study explored the influencing factors of OS and CSS in SCCB patients. Age, marital status, AJCC stage, T stage, M stage, procedure, surgical approach, chemotherapy, tumor size, and lung metastasis were independent risk factors affecting patients' OS. However, age, marital status, T stage, M stage, surgery, chemotherapy, tumor size, lung metastasis, and distant lymph node metastasis were the independent risk factors affecting the CSS in the patients. Based on the above data, we developed nomograms for predicting OS and CSS in SCCB patients. The internal cross-validation showed that the nomograms have excellent accuracy and reliability, which can help us make clinically assisted decisions.

## Supplementary Information

Supplementary Information 1.

Supplementary Information 2.

## Data Availability

The SEER data analyzed in this study is available at https://seer.Cancer.gov/.

## References

[1] Richters A, Aben KKH, Kiemeney LALM (2020). The global burden of urinary bladder cancer: An update. World J. Urol..

[2] Sung H, Ferlay J, Siegel RL, Laversanne M, Soerjomataram I, Jemal A, Bray F (2021). Global cancer statistics 2020: GLOBOCAN estimates of incidence and mortality worldwide for 36 cancers in 185 countries. CA Cancer J. Clin..

[3] Abrahams NA, Moran C, Reyes AO, Siefker-Radtke A, Ayala AG (2005). Small cell carcinoma of the bladder: A contemporary clinicopathological study of 51 cases. Histopathology.

[4] Grignon DJ, Ro JY, Ayala AG, Shum DT, Ordóñez NG, Logothetis CJ, Johnson DE, Mackay B (1992). Small cell carcinoma of the urinary bladder: A clinicopathologic analysis of 22 cases. Cancer.

[5] Bex A, Nieuwenhuijzen JA, Kerst M, Pos F, van Boven H, Meinhardt W, Horenblas S (2005). Small cell carcinoma of bladder: A single-center prospective study of 25 cases treated in analogy to small cell lung cancer. Urology.

[6] Balachandran VP, Gonen M, Smith JJ, DeMatteo RP (2015). Nomograms in oncology: More than meets the eye. Lancet Oncol..

[7] Wang J, Wu Y, He W, Yang B, Gou X (2020). Nomogram for predicting overall survival of patients with bladder cancer: A population-based study. Int. J. Biol. Markers..

[8] Huang C, Zhou W, Song P, Yuan N (2019). Comparison of different prognostic models for predicting cancer-specific survival in bladder transitional cell carcinoma. Future Oncol..

[9] Dong F, Shen Y, Gao F, Shi X, Xu T, Wang X, Zhang X, Zhong S, Zhang M, Chen S, Shen Z (2018). Nomograms to predict individual prognosis of patients with primary small cell carcinoma of the bladder. J. Cancer..

[10] Naito A, Taguchi S, Nakagawa T, Matsumoto A, Nagase Y, Tabata M, Miyakawa J, Suzuki M, Nishimatsu H, Enomoto Y, Takahashi S, Okaneya T, Yamada D, Tachikawa T, Minowada S, Fujimura T, Fukuhara H, Kume H, Homma Y (2017). Prognostic significance of serum neuron-specific enolase in small cell carcinoma of the urinary bladder. World J. Urol..

[11] Howard FM, Pearson AT (2020). Prognosis and treatment of non-small cell lung cancer in the age of deep learning. JAMA Netw. Open..

[12] Liu N, Johnson KJ, Ma CX (2018). Male breast cancer: An updated surveillance, epidemiology, and end results data analysis. Clin. Breast Cancer..

[13] Kamat AM, Hahn NM, Efstathiou JA, Lerner SP, Malmström PU, Choi W, Guo CC, Lotan Y, Kassouf W (2016). Bladder cancer. Lancet.

[14] Feng H, Zhang W, Li J, Lu X (2015). Different patterns in the prognostic value of age for bladder cancer-specific survival depending on tumor stages. Am. J. Cancer Res..

[15] Linn JF, Sesterhenn I, Mostofi FK, Schoenberg M (1998). The molecular characteristics of bladder cancer in young patients. J. Urol..

[16] Koay EJ, Teh BS, Paulino AC, Butler EB (2011). A Surveillance, epidemiology, and end results analysis of small cell carcinoma of the bladder: Epidemiology, prognostic variables, and treatment trends. Cancer.

[17] Chen ZH, Yang KB, Zhang YZ, Wu CF, Wen DW, Lv JW, Zhu GL, Du XJ, Chen L, Zhou GQ, Liu Q, Sun Y, Ma J, Xu C, Lin L (2021). Assessment of modifiable factors for the association of marital status with cancer-specific survival. JAMA Netw. Open..

[18] Tao L, Pan X, Zhang L, Wang J, Zhang Z, Zhang L, Liang C (2020). Marital status and prognostic nomogram for bladder cancer with distant metastasis: A SEER-based study. Front. Oncol..

[19] Sammon JD, Morgan M, Djahangirian O, Trinh QD, Sun M, Ghani KR, Jeong W, Jhaveri J, Ehlert M, Schmitges J, Bianchi M, Shariat SF, Perrotte P, Rogers CG, Peabody JO, Menon M, Karakiewicz PI (2012). Marital status: A gender-independent risk factor for poorer survival after radical cystectomy. BJU Int..

[20] Babjuk M, Burger M, Zigeuner R, Shariat SF, van Rhijn BW, Compérat E, Sylvester RJ, Kaasinen E, Böhle A, Palou Redorta J, Rouprêt M, European Association of Urology (2013). EAU guidelines on non-muscle-invasive urothelial carcinoma of the bladder: Update 2013. Eur. Urol..

[21] Lee A, Lee HJ, Huang HH, Ho H, Chen K (2019). Low-risk non-muscle-invasive bladder cancer: Further prognostic stratification into the "very-low-risk" group based on tumor size. Int. J. Urol..

[22] Tully KH, Moschini M, von Rundstedt FE, Aziz A, Kluth LA, Necchi A, Rink M, Hendricksen K, Sargos P, Vetterlein MW, Seiler R, Poyet C, Krajewski W, Fajkovic H, Shariat SF, Xylinas E, Roghmann F (2020). Impact of tumor size on the oncological outcome of high-grade nonmuscle invasive bladder cancer: Examining the utility of classifying Ta bladder cancer based on size. Urol. Oncol..

[23] Tian Z, Meng L, Wang X, Diao T, Hu M, Wang M, Zhang Y, Liu M (2021). Predictive nomogram and risk factors for lymph node metastasis in bladder cancer. Front. Oncol..

[24] Zhan X, Jiang M, Deng W, Liu X, Chen L, Fu B (2022). Development and validation of a prognostic nomogram for predicting cancer-specific survival in patients with lymph node positive bladder cancer: A study based on SEER database. Front. Oncol..

[25] Cattrini C, Cerbone L, Rubagotti A, Zinoli L, Latocca MM, Messina C, Zanardi E, Boccardo F (2019). Prognostic variables in patients with non-metastatic small-cell neuroendocrine carcinoma of the bladder: A population-based study. Clin. Genitourin. Cancer..

[26] Pasquier D, Barney B, Sundar S, Poortmans P, Villa S, Nasrallah H, Boujelbene N, Ghadjar P, Lassen-Ramshad Y, Senkus E, Oar A, Roelandts M, Amichetti M, Vees H, Zilli T, Ozsahin M (2015). Small cell carcinoma of the urinary bladder: A retrospective, multicenter rare cancer network study of 107 patients. Int. J. Radiat. Oncol. Biol. Phys..

[27] Thota S, Kistangari G, Daw H, Spiro T (2013). A clinical review of small-cell carcinoma of the urinary bladder. Clin. Genitourin. Cancer..

[28] Mackey JR, Au HJ, Hugh J, Venner P (1998). Genitourinary small cell carcinoma: Determination of clinical and therapeutic factors associated with survival. J. Urol..

[29] Chau C, Rimmer Y, Choudhury A, Leaning Frcr D, Law A, Enting D, Lim JH, Hafeez S, Khoo V, Huddart R, Mitchell D, Henderson DR, McGrane J, Beresford M, Vasudev N, Beesley S, Hilman S, Manetta C, Sriram R, Sharma A, Eswar C, Treece S, Vilarino-Varela M, Varughese M, Glen H, Pintus E, Crabb S (2021). Treatment outcomes for small cell carcinoma of the bladder: Results from a UK patient retrospective cohort study. Int. J. Radiat. Oncol. Biol. Phys..

[30] Yang Z, Bai Y, Liu M, Hu X, Han P (2020). Development and validation of a prognostic nomogram for predicting cancer-specific survival after radical cystectomy in patients with bladder cancer: A population-based study. Cancer Med..

[31] Yang Z, Bai Y, Liu M, Hu X, Han P (2022). Development and validation of prognostic nomograms to predict overall and cancer-specific survival for patients with adenocarcinoma of the urinary bladder: A population-based study. J. Invest. Surg..

