# Supplementary material for: Surveillance of prognostic risk factors in patients with SCCB using artificial intelligence: a retrospective study
Source: Sci Rep. 2023 May 30;13:8727. doi: 10.1038/s41598-023-35761-w (PMC10229646; doi:10.1038/s41598-023-35761-w)
Supplement: Supplementary file 1 — Supplementary Information 1. [file 41598_2023_35761_MOESM1_ESM.docx]

**Figure S1.** The number of bladder cancer patients and the ratio of SCCB.
