# Supplementary figures and images for: Surveillance of prognostic risk factors in patients with SCCB using artificial intelligence: a retrospective study
Source: Sci Rep. 2023 May 30;13:8727. doi: 10.1038/s41598-023-35761-w (PMC10229646; doi:10.1038/s41598-023-35761-w)

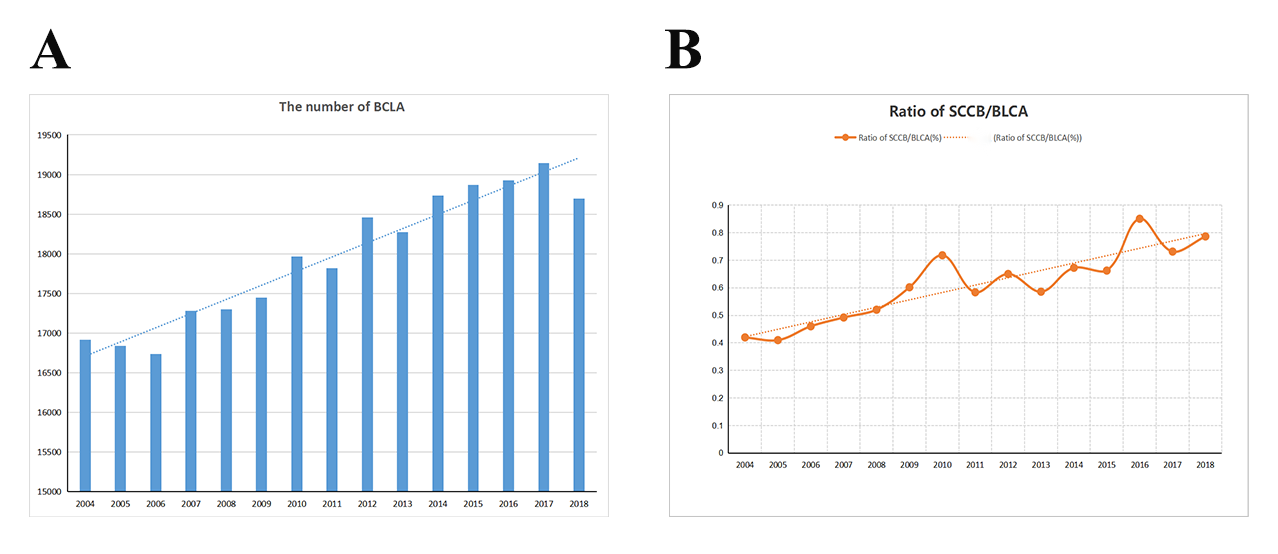

Supplement: Supplementary file 2 — Supplementary Information 2. [file 41598_2023_35761_MOESM2_ESM.png]
